# Predictors of Diet-Induced Weight Loss in Overweight Adults with Type 2 Diabetes

**DOI:** 10.1371/journal.pone.0160774

**Published:** 2016-08-05

**Authors:** Kirsten A. Berk, Monique T. Mulder, Adrie J. M. Verhoeven, Herman van Wietmarschen, Ruud Boessen, Linette P. Pellis, Adriaan van t Spijker, Reinier Timman, Behiye Ozcan, Eric J. G. Sijbrands

**Affiliations:** 1 Department of Internal Medicine, Section Pharmacology Vascular and Metabolic diseases, Erasmus Medical Center, Rotterdam, The Netherlands; 2 Department of Psychiatry, unit of Medical Psychology and Psychotherapy, Erasmus Medical Center, Rotterdam, The Netherlands; 3 Department of Microbiology & Systems Biology, TNO Zeist, Zeist, The Netherlands; Children's National Health System, UNITED STATES

## Abstract

**Aims:**

A very low calorie diet improves the metabolic regulation of obesity related type 2 diabetes, but not for all patients, which leads to frustration in patients and professionals alike. The aim of this study was to develop a prediction model of diet-induced weight loss in type 2 diabetes.

**Methods:**

192 patients with type 2 diabetes and BMI>27 kg/m^2^ from the outpatient diabetes clinic of the Erasmus Medical Center underwent an 8-week very low calorie diet. Baseline demographic, psychological and physiological parameters were measured and the C-index was calculated of the model with the largest explained variance of relative weight loss using backward linear regression analysis. The model was internally validated using bootstrapping techniques.

**Results:**

Weight loss after the diet was 7.8±4.6 kg (95%CI 7.2–8.5; p<0.001) and was independently associated with the baseline variables fasting glucose (B = -0.33 (95%CI -0.49, -0.18), *p* = 0.001), anxiety (HADS; B = -0.22 (95%CI -0.34, -0.11), *p* = 0.001), numb feeling in extremities (B = 1.86 (95%CI 0.85, 2.87), *p* = 0.002), insulin dose (B = 0.01 (95%CI 0.00, 0.02), *p* = 0.014) and waist-to-hip ratio (B = 6.79 (95%CI 2.10, 11.78), *p* = 0.003). This model explained 25% of the variance in weight loss. The C-index of this model to predict successful (≥5%) weight loss was 0.74 (95%CI 0.67–0.82), with a sensitivity of 0.93 (95% CI 0.89–0.97) and specificity of 0.29 (95% CI 0.16–0.42). When only the obese T2D patients (BMI≥30 kg/m^2^; n = 181) were considered, age also contributed to the model (B = 0.06 (95%CI 0.02, 0.11), *p* = 0.008), whereas waist-to-hip ratio did not.

**Conclusions:**

Diet-induced weight loss in overweight adults with T2D was predicted by five baseline parameters, which were predominantly diabetes related. However, failure seems difficult to predict. We propose to test this prediction model in future prospective diet intervention studies in patients with type 2 diabetes.

## Introduction

The metabolic regulation of obesity-related type 2 diabetes improves with diet-induced weight loss. A very low calorie diet is successful in that respect, but unfortunately not in all patients, which leads to feelings of failure and reduction of cost-effectiveness of the treatment. A prediction model of weight loss can assist selecting those individuals that will benefit most.

The dramatic rise in the world-wide prevalence of obesity has led to a rising prevalence of T2D. It is estimated that 82–87% of the T2D population are overweight or obese [1]. In patients with T2D, a high BMI and waist circumference are associated with increased mortality [2,3]. Conversely, moderate weight loss improves glycaemic control, lipid profile and blood pressure in these individuals and has been associated with reduced mortality [4–6]. Therefore, weight loss is an important aspect of treatment of T2D. Weight loss of more than 5% results in important health benefits and reduces health care costs [7,8]. However, not all individuals that are overweight or obese achieve and maintain 5% weight loss or more with lifestyle interventions [6,9]. Predictors of weight loss may be very helpful in optimizing individualized weight loss strategies and in selecting those individuals who will benefit most from a diet, ultimately improving treatment outcome and reducing health care costs. In adults with obesity, a number of physiological and psychological predictors of weight loss have been identified. These include sex, previous dieting for weight loss, initial weight, motivation, self-efficacy, self-esteem and exercise [10,11]. However, these predictors of weight loss have not been studied in subjects with T2D, and diabetes-specific variables have not been included in previous research in this field. Both psychological and physiological factors are most probably of importance, as obesity-related T2D is a complex multifactorial disease.

Therefore, the purpose of the present study was to identify which factors predict diet-induced weight loss in overweight and obese adults with T2D, using psychological, physiological as well as diabetes-related variables.

## Materials and Methods

We enrolled participants of the run-in phase of the Prevention Of Weight Regain (POWER) trial of which the protocol has been published previously [12]. This study was approved by the Medical Ethics Committee of the Erasmus Medical Center in Rotterdam (reference number MEC-2009-143/NL26508.078.09), in compliance with the Helsinki Declaration. All participants provided written informed consent before participating in this study.

### Study population

Patients with T2D having BMI>27 kg/m^2^, whose age was 18–75 years, were recruited from the outpatient diabetes clinic of the Erasmus Medical Center, Rotterdam from 2010–2013. Exclusion criteria were pregnancy, lactation, severe psychiatric problems, significant cardiac arrhythmias, unstable angina, decompensated congestive heart failure, major organ system failure, untreated hypothyroidism, and end-stage renal disease. Patients who had a myocardial infarction, cerebrovascular accident or major surgery during the previous 3 months were also excluded.

### Diet intervention and data collection

#### Intervention

After signing informed consent, participants followed a Very Low Calorie Diet (VLCD) for a period of 8 weeks. The VLCD consisted of 2 commercially available meal replacements per day (Glucerna SR^®^, Abbott Nutrition BV), plus 75 grams of lean meat, one skimmed milk product and vegetables. Daily intake was approximately 750 kcal. Participants received oral and written instructions on how to follow this diet at home. Before the start of the diet the anti-diabetes medication was lowered to prevent hypoglycaemia: oral anti-diabetic agents (except metformin) and short-acting insulin analogues were discontinued, while the doses of long-acting insulin analogues and biphasic mixtures were halved. GLP-1 analogues and DPP-4 inhibitors were continued. One week after starting the diet, participants received a phone call of the research team. A structured discussion about blood glucose measurements, questions about the diet, problems with adherence to the diet, and complaints was performed. Participants were encouraged to follow the dietary recommendations of the study and to weekly email their blood glucose measurements. The insulin dose was adjusted during the diet period based on these daily glucose measurements.

#### Measures and outcomes

The primary outcome was weight loss, expressed as a percentage of baseline bodyweight. At baseline and at the end of the diet intervention bodyweight was measured. Weight loss was considered ‘successful’ if ≥5% reduction of baseline body weight was achieved. Moderate weight loss of ≥5% is considered by the American Diabetes Association and the European Association of the Study of Diabetes to produce significant health benefits [13,14].

Besides age and sex, the baseline variables were as follows:

1. Weight and BMI
  Weight was measured to the nearest 0.1 kg using the same Seca 888 compact digital flat scale after removal of shoes. Height was measured to the nearest 0.5 cm without shoes using a Seca stadiometer.
2. Waist circumference and waist-to-hip ratio
  Waist circumference (cm) was measured at the level midway between the lowest rib margin and the aliac crest. Hip circumference was measured at the widest point over the buttocks. Both waist- and hip circumference were measured by the nearest 0,5 cm, using a tape-measure. Subsequently, waist-hip ratio (WHR) was calculated.
3. Glycemic control
  - Fasting glucose, measured using standard laboratory techniques
  - Hemoglobin A1c (HbA1c), measured using standard laboratory techniques
  - Total daily insulin dose (IU/day)
  - Total daily metformin dose (mg/day)
4. Physical complaints related to diabetes
  - Polyuria (yes/no)
  - Pruritus (yes/no)
  - Eye problems (yes/no)
  - Numbness in extremities (yes/no)
5. Exercise
  Days per week ≥30 minutes of exercise
6. History of dieting for weight loss
  Number of weight loss attempts
7. Fatigue
  Measured by the Checklist Individual Strength (CIS) [15], which consists of 20 statements for which the respondent has to indicate on a 7-point scale (from true to false) to what extent the particular statement applies to him or her. The statements refer to four fatigue aspects: (1) subjective fatigue (2) reduced motivation (3) reduced activity and (4) reduced concentration. People with a score above 76 are at increased risk of long-term sickness absence.
8. Depression and anxiety
  Measured by the Hospital Anxiety and Depression scale (HADS). The HADS consists of a 7-item Anxiety scale and a 7-item Depression Scale. Each item is scored from 0 to 3. A total score on either scale below 8 excludes anxiety or depression, respectively. A score of 8–10 and 11–21 indicates a possible and probable anxiety/depression, respectively [16].
9. Quality of life
  Measured by the EQ-5D VAS score, ranging from 0 (death) to 100 (perfect health) [17,18]
10. Self-esteem
  Measured by the Rosenberg Self-esteem Scale (RSE), which is a 10-item questionnaire that measures global self-esteem. Items are scored on a 4-point scale. A higher score indicates a more positive self-esteem. Scores below 21 indicate low self-esteem [19]
11. Eating disorders
  - Measured by the Eating Disorder Examination-Questionnaire (EDE-Q), a 36 item questionnaire that measures concerns about shape, weight and eating, restraint and binge eating. Total EDE-Q score range between 0–6. A higher score indicates more severe eating psychopathology [20].
  - Binge eating disorder (BED), measured by use of the DSM-V criteria (yes/no)

All data were filed in a database using a trial management system (OpenClinica^®^).

### Statistical Analysis

Variables at baseline were expressed as number with percentage or mean with standard deviation. Prior to analysis, missing data were imputed using regression imputation. Participants with more than 50% missing data were excluded. For 7 participants, whose body weight at the end of the diet intervention was not available due to attrition, we assumed 0% weight loss. We analysed the data according to the intention-to-treat as well as the per-protocol principle, leaving those 7 patients out of the analysis. The average weight loss was analysed using a paired samples t-test. The difference between groups at baseline was tested with a Chi-Square test, an independent samples t-test or a Mann-Whitney U test, depending on normality of the data. Potential predictors were identified by univariate regression analyses with percentage weight loss as dependent variable. Subsequently, all variables were entered into a multiple regression analysis. The least informative covariates were successively removed from the model in a backward stepwise elimination procedure based on the Akaike information criterion (AIC)[21]. We accepted a maximum Variance Inflation Factor of 5 to exclude multicollinearity problems. Each step of the backward regression analysis was internally validated using bootstrapping [22]. In short, 1000 bootstrap cohorts (of equal size as the original dataset) were randomly drawn, with replacement, from the cases in the original dataset. Logistic regression was applied to predict successful (≥ 5% = 1) and unsuccessful (< 5% = 0) weight loss. The relationship between false positives (1—specificity) and true positives (sensitivity) of this prediction was presented in a ROC (Receiving Operating Characteristic) curve. For the dichotomized prediction, the sensitivity, specificity and positive predictive value were calculated. Statistical significance was set at p < 0.05. All data processing and analyses were carried out in SPSS (version 21).

## Results

**Fig 1:**
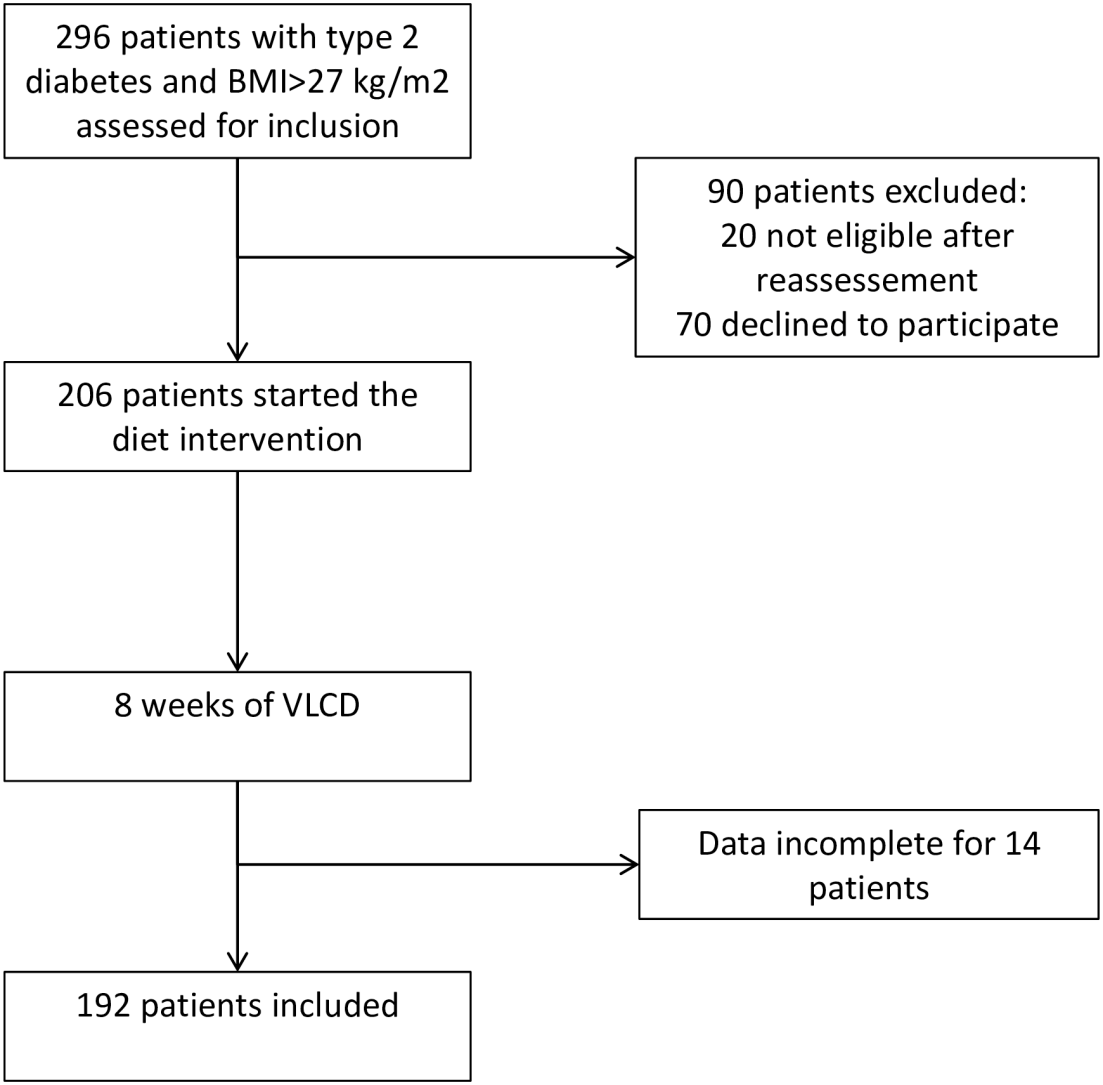
Study flow chart.

From March 2010 to May 2013, we assessed 296 patients with BMI>27 kg/m^2^ and T2D from our out-patient diabetes clinic, of whom 276 were eligible and 206 were willing to participate in this study (Fig 1). Individuals who declined to participate were older (58.0 (49.8–64.0) years vs. 53.5 (47.0–61.8) years, *p* = 0.04) and more often male (61% vs. 42%, *p* = 0.005) than the individuals who were willing to participate in this study. Ethnicity did not differ. The main reasons for the declined participation were work related and lack of time. Fourteen participants did not fill out the questionnaires and did not provide blood samples, hence we collected data from 192 participants.

Table 1 shows baseline characteristics of our study population, of which 58% were female. Of the participants, 181 (94.3%) had a BMI>30 kg/m^2^, thus qualified as obese. Mean HbA1c and fasting glucose levels were well above target levels despite antidiabetic medication. Sixty-two percent of the participants used insulin (62.0±63.9 IU/day). According to the HADS norm, 18% had a clinical depression and 20% a clinical anxiety disorder. Twelve participants (6.3%) used antidepressants (self-reported).

**Table 1:** General baseline characteristics (n = 192).

Characteristics	Value, mean ± SD (range)
Age (y)	54.2 ± 10.6 (26–75)
Sex, female (n (%))	112 (58%)
Exercise (weekly days ≥ 30 min. exercise)	5.3 ± 2.3 (0–7)
*Physiological variables*:	
Weight (kg)	106.0 ± 19.0 (69.4–169.2)
BMI (kg/m^2^)	37.0 ± 5.6 (27.3–62.6)
Waist circumference (cm)	120.2 ± 13.3 (90–163)
Waist-to-hip ratio	1.0 ± 0.1 (0.8–1.3)
HbA1c (mmol/mol)	63.9 ± 15.2 (32–126)
HbA1c (%)	8.0 ± 1.4 (5.1–13.7)
Fasting glucose (mmol/l)	9.5 ± 3.2 (3.2–21.1)
Insulin dose (IU)^a^ and users (n (%))	100.1 ± 52.7 (8–248) 119 (62%)
Metformin dose (mg)^a^ and users (n (%))	1774.8 ±754.2 (500–3000) and users (n (%))
Numb feeling in extremities (n (%))	96 (50%)
Polyuria (n (%))	57 (30%)
Pruritus (n (%))	37 (19%)
Diabetes-related eye problems (n (%))	45 (23%)
*Psychological variables*:	
Depression score (HADS 0–20)	7.0 ± 4.0 (0–20)
Clinical depression (n (%))	35 (18%)
Anxiety score (HADS 0–20)	6.6 ± 4.3 (0–20)
Clinical anxiety disorder (n (%))	38 (20%)
Self-esteem (RSE 0–40)	30.6 ± 5.6 (13–40)
Quality of life (EQ5D VAS 1–100)	58.8 ± 21.0 (10–100)
Fatigue (CIS total score 20–140)	80.9 ± 23.8 (25–133)
Eating disorders (EDE-Q total score 0–6)	2.2 ± 1.2 (0–5.4)
Binge eating disorder (DSM V, n (%))	11 (6%)
Diet history (no. of weight loss attempts)	1.0 ± 1.1 (0–5)

^a^dose only in users

After 8 weeks of VLCD, participants had lost 7.8±4.6 kg (95%CI 7.2–8.5; *p*<0.001). Twenty-five percent of the participants lost less than 5%, 28% lost 5–7.5%, 21% lost 7.5–10% and 26% lost more than 10% of their bodyweight. Compared with the successful weight loss group, the unsuccessful weight loss group (i.e. <5%) showed higher levels of HbA1c and fasting glucose, and were more likely to have pruritus and a lower self-esteem (*p*<0.01).

In univariate regression analyses (Table 2), age, waist-to-hip ratio, numb feeling in extremities, and self-esteem were all positively associated with relative weight loss, while female sex, fasting glucose, HbA1c, pruritus and anxiety were negatively associated with relative weight loss.

**Table 2:** Univariate linear regression analysis of relative weight loss with baseline variables.

Covariate	B	SE	β	*p*
Age (y)	0.086	0.026	0.237	**0.001**
Sex (female)	-1.908	0.547	-0.246	**0.001**
Exercise (weekly days ≥ 30 min. exercise)	0.137	0.119	0.083	0.251
*Physiological variables*:				
Weight (kg)	0.014	0.015	0.071	0.325
Waist circumference (cm)	0.040	0.021	0.137	0.058
Waist-to-hip ratio	10.160	2.927	0.244	**0.001**
Fasting glucose (mmol/l)	-0.311	0.085	-0.257	**<0.001**
HbA1c (mmol/mol)	-0.046	0.018	-0.181	**0.012**
Insulin dose (IU)	0.008	0.004	0.131	0.070
Insulin dose change during diet (IU)	-0.019	0.006	-0.245	**0.001**
Metformin dose (mg)	<0.0001	<0.0001	0.007	0.922
Numbness in extremities (n = 0/y = 1)	1.792	0.540	0.234	**0.001**
Polyuria (n = 0/y = 1)	0.019	0.608	0.002	0.976
Pruritus (n = 0/y = 1)	-1.732	0.693	-0.178	**0.013**
Diabetes-related eye problems (n = 0/y = 1)	-0.648	0.654	-0.072	0.324
*Psychological variables*:				
Depression score (HADS 0–20)	-0.132	0.070	-0.136	0.060
Anxiety score (HADS 0–20)	-0.223	0.062	-0.252	**<0.001**
Self-esteem (RSE 0–40)	0.126	0.049	0.184	**0.011**
Quality of life (EQ5D VAS 1–100)	0.024	0.013	0.131	0.071
Fatigue (CIS total score 20–140)	-0.011	0.012	-0.065	0.371
Eating disorders (EDE-Q total score 0–6)	-0.276	0.238	-0.084	0.247
Binge eating disorder (DSM V, n = 0/y = 1)	0.049	1.189	0.003	0.967
Diet history (no. of weight loss attempts)	-0.111	0.248	-0.032	0.656

**Fig 2:**
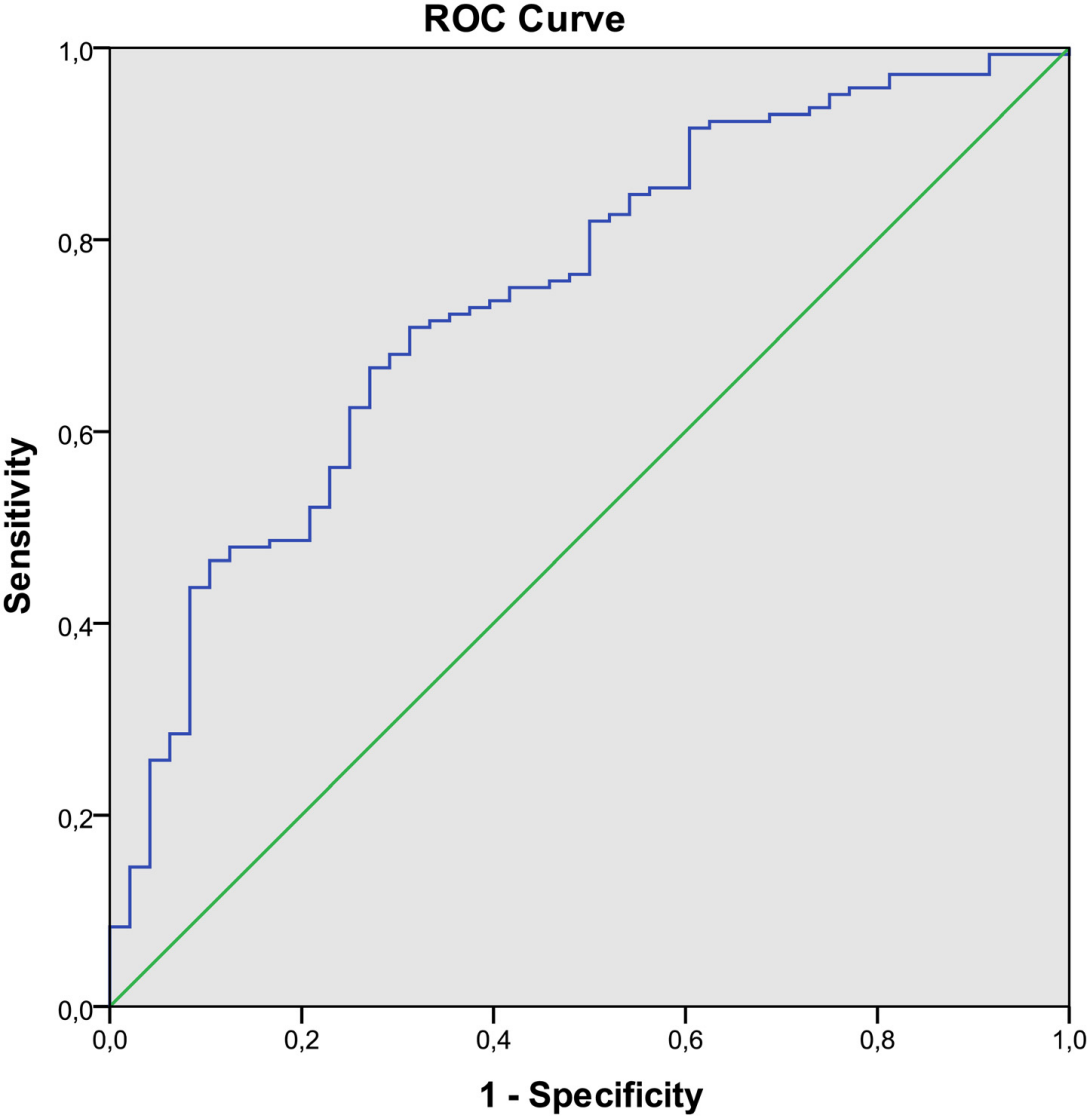
ROC Curve of multiple logistic regression model of successful relative weight loss (≥5%). Variables included in the multiple linear regression model: fasting glucose level, insulin dose, waist-to-hip ratio, numb feeling in extremities and anxiety score.

Table 3 shows the results of the multiple linear regression analysis. Since both fasting glucose and HbA1c, and waist circumference and waist-to-hip ratio were strongly correlated (*p*<0.001), we included only fasting glucose and waist-to-hip ratio in the multivariate analysis to avoid multicollinearity problems. In the backward stepwise elimination multiple regression analysis (using bootstrapping), the final model contained 5 baseline variables which were independently associated with relative weight loss: the higher the reported numb feeling in extremities (B = 1.86 (95%CI 0.85, 2.87), *p* = 0.002), insulin dose (B = 0.01 (95%CI 0.00, 0.02), *p* = 0.014) and waist-to-hip ratio (B = 6.79 (95%CI 2.10, 11.78), *p* = 0.003), and the lower the fasting glucose level (B = -0.33 (95%CI -0.49, -0.18), *p* = 0.001) and anxiety score (HADS; B = -0.22 (95%CI -0.34, -0.11), *p* = 0.001), the greater the relative weight loss. These five parameters together explained 25% of the variance of relative weight loss (R^2^ = 0.25, *F*(5) = 12.54, *p*<0.001). In Fig 2, we show the ROC curve of our intention-to-treat model in discriminating unsuccessful (<5%) from successful (≥5%) weight loss, which had a C-index of 0.74 (95%CI 0.67–0.82). The logistic model had 0.80 (95% CI 0.74–0.86) post-test likelihood of predicting successful weight loss and 0.58 (95% CI 0.39–0.78) post-test likelihood of predicting unsuccessful weight loss. The model yielded 134 true positive, 34 false positive, 10 false negative and 14 true negative predictions. Hence, the sensitivity of this model was 0.93 (95% CI 0.89–0.97) and the specificity was 0.29 (95% CI 0.16–0.42). When we corrected this model for change in insulin dose during the diet, the five baseline variables remained significantly associated with relative weight loss; the model now explained 30% of the variance of weight loss (R^2^ = 0.30, *F*(6) = 13.31, *p*<0.001) and discriminated successful from unsuccessful weight loss with a C-index of 0.75. However, the VIF statistics for insulin dose and insulin change was 7, indicating possible multicollinearity problems.

**Table 3:** Multiple linear regression model (backward stepwise elimination using bootstrapping) of relative weight loss with baseline variables.

Variable	B	95%CI	95%CI	R^2^	*p*
		Lower	Upper		
Fasting glucose (mmol/l)	-0.332	-0.485	-0.180	0.078	0.001
Insulin dose (IU)	0.010	0.002	0.018	0.032	0.014
Waist-to-hip ratio	6.790	2.096	11.775	0.025	0.003
Numbness in extremities	1.860	0.853	2.874	0.065	0.002
Anxiety (HADS)	-0.219	-0.344	-0.111	0.056	0.001

Explained variance in this model R^2^ = 0.25, *F*(5) = 12.54, *p*<0.001; C-index = 0.74. Variables entered into the model: age; sex; weight; waist-to-hip ratio; fasting glucose; total daily insulin dose; total daily metformin dose; physical complaints related to diabetes (polyuria, pruritus, eye problems, numbness in extremities, all yes/no); exercise (days per week ≥30 minutes of exercise); history of weight loss dieting (number of different diets); fatigue (Checklist Individual Strength); depression and anxiety (Hospital Anxiety and Depression scale); quality of life (EQ-5D VAS score); self-esteem (Rosenberg self-esteem); eating disorders (EDE-Q); and binge eating disorder (DSM-V criteria, yes/no).

We conducted the multivariate regression analysis in a backward stepwise manner, to obtain the optimal model. Alternatively, putting all the univariately associated variables (*p*<0.1) in the multivariate regression model only marginally affected the performance of the model (R^2^ = 0.29, *F*(11) = 6.53, *p*<0.001; C-index = 0.77).

When we excluded the 7 participants who did not finish the diet intervention (per protocol analysis), the model slightly improved, as indicated by the additional contribution of the variables pruritus and quality of life (EQ5D VAS). The lower the reported pruritus and the higher the perceived quality of life, the greater was the weight loss. This model explained 30% of the variance of relative weight loss (R^2^ = 0.30, *F*(8) = 9.29, *p*<0.001) and discriminated successful from unsuccessful weight loss with a C-index of 0.79.

We also performed the analysis for the obese subgroup (BMI≥30 kg/m^2^; n = 181) only. Compared to the entire study population, age now also contributed to the model (B = 0.06 (95%CI 0.02, 0.11), *p* = 0.008), whereas waist-to-hip ratio was removed from the model in the backward elimination procedure. The model containing fasting glucose, insulin dose, age, numb feeling in extremities and anxiety score explained 25% of the variance of relative weight loss (R^2^ = 0.25, *F*(5) = 11.84, *p*<0.001), and discriminated successful from unsuccessful weight loss with a C-index of 0.73.

## Discussion

In this pragmatic explorative study, we found that successful diet-induced weight loss can be predicted in overweight adults with T2D by the baseline variables fasting glucose, anxiety, numb feeling in extremities, insulin dose and waist-to-hip ratio. However, failure seems more difficult to predict. Surprisingly, the ‘usual suspects’ in predicting weight loss in individuals with obesity but without T2D, such as initial weight, diet history, self-esteem and exercise, did not contribute to the model. Remarkably, 3 out of 5 predictors in the model were related to diabetes. This suggests that diabetes control is of major importance for overweight and obese patients with T2D, who aim at losing weight, more than factors that are most relevant in non-diabetic persons with obesity.

Our data showed that participants with a higher baseline fasting glucose and, hence, worse diabetes control were less successful in achieving weight reduction. One could speculate that controlling diabetes might require skills that are also important in controlling weight. A recent review by Ahola, et al. found that factors associated with good management of diabetes included individual issues such as self-efficacy, motivation, coping and problem-solving skills, as well as issues related to the environment such as social support and socio-economic factors [23]. All of these factors have been linked with successful weight loss as well [10]. Alternatively, participants with a high fasting glucose may lose energy through glycosuria. Losing weight reduces blood glucose levels in T2D [6] and subsequently the energy deficit due to glycosuria may disappear, leading to a disadvantage in weight loss for participants with a very high fasting glucose at baseline.

We identified anxiety as an unexpected negative predictor for weight loss. Among our participants with T2D, the prevalence of anxiety disorder was as high as 20%. This is higher than the prevalence of anxiety disorder in the Dutch population (7.7% for males and 12.5% for females [24]), and in line with previous studies in T2D [25]. While anxiety has not been extensively studied in the context of weight control, it has been correlated with a defensive coping style: avoiding problems rather than solving them [26]. In diabetes, an anxious, defensive coping style has been associated with decreased adherence to therapy [27]. Similarly, in our study anxiety could have led to a decreased adherence to the diet. A more autonomous coping strategy has been shown to correlate better with the capability to lose weight than a defensive coping style [11,28]. It would be interesting to test whether treating anxiety prior to the diet intervention would improve the success rate of a weight loss diet.

Baseline insulin dose was positively associated with weight loss. Before starting with the VLCD, we reduced the insulin dose by more than 50% to avoid hypoglycaemic events. Insulin use has been associated with weight gain due to the stimulation of lipogenesis and a reduction of lipolysis and glycosuria [29]. Lowering the insulin dose could have had the opposite effect, where participants on a high baseline dose could have had more benefit from the greater absolute reduction of insulin dose. Reducing the insulin dose before a weight loss attempt is important to prevent hypoglycaemia, but it may also enhance weight loss. Indeed, baseline insulin dose and change in insulin dose were highly correlated (r = -0.929, *p*<0.001), so the association of baseline insulin with weight loss could very well have been the effect of lowering the insulin dose. However, when we corrected the model for change in insulin dose, baseline insulin dose was still significantly associated with relative weight loss.

Experiencing a numb feeling in extremities was positively associated with the relative weight loss. Of the participants who lost less than 5% weight, 35% reported this complaint, compared with 55% in the successful weight loss group. A numb feeling in extremities could be associated with diabetic neuropathy. Indeed, numbness was significantly correlated with a history of diabetic neuropathy (data not shown) and therefore probably with the severity of complications. Complications or perceiving more complaints could be a motivational trigger for patients to adhere to the diet regimen, although this has not been studied previously.

Participants with a higher waist-to-hip ratio and to a lesser extent waist circumference, i.e. more abdominal fat, were more prone to lose weight in our study population. Moderate weight loss, particularly by a VLCD, has been shown to result in preferential loss of visceral fat mass [30]. Visceral fat mass is associated with reduced insulin sensitivity and increased systemic low-grade inflammation [2,31]. Men tend to have a higher waist-to-hip ratio than women, as was the case in our population (1.07±0.07 vs. 0.94±0.06, *p*<0.001). Since males have a higher energy expenditure compared to women, and the diet intervention was the same for both men and women, the association we found could be explained by the higher energy deficit for the male participants. Accordingly, the univariate analysis showed that women lost significantly less weight than men, suggesting that men will benefit most from weight reduction and preferential abdominal fat mass loss. However, in the multiple linear regression analysis, sex no longer contributed significantly to weight loss while waist-to-hip ratio remained an independent contributor. Waist-to-hip ratio is a stronger predictor for weight loss success than sex.

A number of covariates, such as baseline bodyweight, self-motivation, diet history and self-esteem have previously been associated with diet-induced weight loss in individuals with obesity [10,11]. However, they did not predict relative weight loss in the present study. Self-esteem was positively associated with weight loss in the univariate analysis, but no longer in the multiple analysis. In concordance with most other studies on predictors of weight loss in subjects with obesity [10,32], binge eating disorders and baseline exercise behaviour were not associated with weight loss. In previous studies, depression or other measures of psychological well-being were not predictive for weight loss [33], as was the case in the present study, or were negatively associated with weight loss [34].

### Limitations and Strengths

The present study is a pragmatic and explorative before-after study in the run-in phase of a trial. We internally validated our results using statistical techniques (bootstrapping), however, future prospective replication studies are required in order to confirm our findings. With the baseline variables that were included in our study, 25% of the variance of weight loss could be explained, leaving a large part unexplained. Future studies could build further on the model presented here in an independent patient cohort, including other possible predictors like plasma hormone levels or genomic profiles. A limitation of this study is the relatively low number of participants with unsuccessful weight loss, diminishing the discriminating power of the model. Due to the academic hospital setting, our study population may not be representative of the general population with T2D and overweight. However, a substantial percentage of our population had been referred to us by the general practitioner with participation in our study as exclusive purpose, leading to a relatively heterogeneous patient cohort in a ‘real life’ clinical setting. Finally, we studied the short-term effect of the diet intervention only. However, a relatively quick success of the intervention in terms of weight loss and improved glycemic control may motivate the patients with obesity and diabetes to change lifestyle necessary to achieve long term health benefit. Additional studies are required to determine whether the model also predicts long-term maintenance of weight loss. Strengths of the current study are the prospective design, the relative large cohort of patients with T2D, the low attrition rate (3%) and the consideration of psychological as well as physiological variables.

### Conclusion and clinical implications

The current study suggests that diet-induced weight loss can be predicted by five easily measurable psychological and physiological variables, with a positive post-test probability of 80%. Diabetes specific variables were better at predicting successful weight loss during a VLCD than the predictors known from non-T2D obese cohorts. Future prospective studies in the T2D population are needed in order to replicate these findings. Restricting a VLCD to individuals with a high post-test likelihood of successful weight loss seems attractive as it might increase efficacy and improve the cost-effectiveness. Such screening of patients and predicting their treatment success will bring us one step closer to personalized treatment of diabetes.
